# Supplementary material for: Sharing GWAS summary statistics results in more citations
Source: Commun Biol. 2023 Jan 28;6:116. doi: 10.1038/s42003-023-04497-8 (PMC9884206; doi:10.1038/s42003-023-04497-8)
Supplement: Supplementary file 1 — Description of Additional Supplementary Files [file 42003_2023_4497_MOESM1_ESM.pdf]

Description of Additional Supplementary Files

File name: Supplementary Data 1
Description: Studies and summary statistics datasets included in this study.

File name: Supplementary Data 2
Description: Analysis of articles published on Nature Genetics or PLoS Genetics without summary data on GWAS catalog.

File name: Supplementary Data 3
Description: Articles without GWAS summary statistics in GWAS catalog, with updated classification.

File name: Supplementary Data 4
Description: Results for logistic model of effects on sharing.

File name: Supplementary Data 5
Description: Results for linear model of effects on citations (measured as log(RCR)).

File name: Supplementary Data 6
Description: GWAS-publishing journals in GWAS catalog with SJR data (2007 - 2020), including those that shared summary statistics in GWAS catalog and/or elsewhere. Journals selected to analyse their individual effects highlighted in bold.
